# Pigeons at the edge of the empire: Bioarchaeological evidences for extensive management of pigeons in a Byzantine desert settlement in the southern Levant

**DOI:** 10.1371/journal.pone.0193206

**Published:** 2018-03-21

**Authors:** Nimrod Marom, Baruch Rosen, Yotam Tepper, Guy Bar-Oz

**Affiliations:** 1 Laboratory of Archaeozoology, Institute of Archaeology, University of Haifa, Haifa, Israel; 2 Agricultural Research Organization, Bet Dagan, Israel; Institut Català de Paleoecologia Humana i Evolució Social (IPHES), SPAIN

## Abstract

Metric data of 6^th^ century CE pigeons from the Negev Desert, Israel, are employed to test competing hypotheses on flock management strategies: that directed selection for size or shape took place under intensive management; or, alternatively, that stabilizing selection was a stronger determinant of size and shape under extensive management conditions. The results of the analysis support the second hypothesis by demonstrating that the Byzantine Negev pigeons were like wild pigeon (*Columba livia*) in shape, albeit small-sized. The inferred extensive management system is then discussed in the context of pigeon domestication and human micro-ecologies in marginal regions.

## Introduction

The importance of pigeon breeding in the study of evolution [1] contrasts with the limited empirical knowledge on pigeon keeping in antiquity. Human-pigeon interaction until the modern period is currently glimpsed through scattered historical anecdotes from the Roman world, e.g. by Pliny [2] and Columella [3], on works of art [4] and on columbaria. Bioarchaeological evidences bearing on the subject are almost nonexistent, the result of poor preservation of the fragile bird bones in archaeological sediments and size-related recovery bias in excavations. The rare pigeon bones found intermittently in archaeological finds dating from the Pleistocene [5] to the Roman period [6] are isolated specimens that do not provide information on population variables such as age and size distributions.

**Fig 1:**
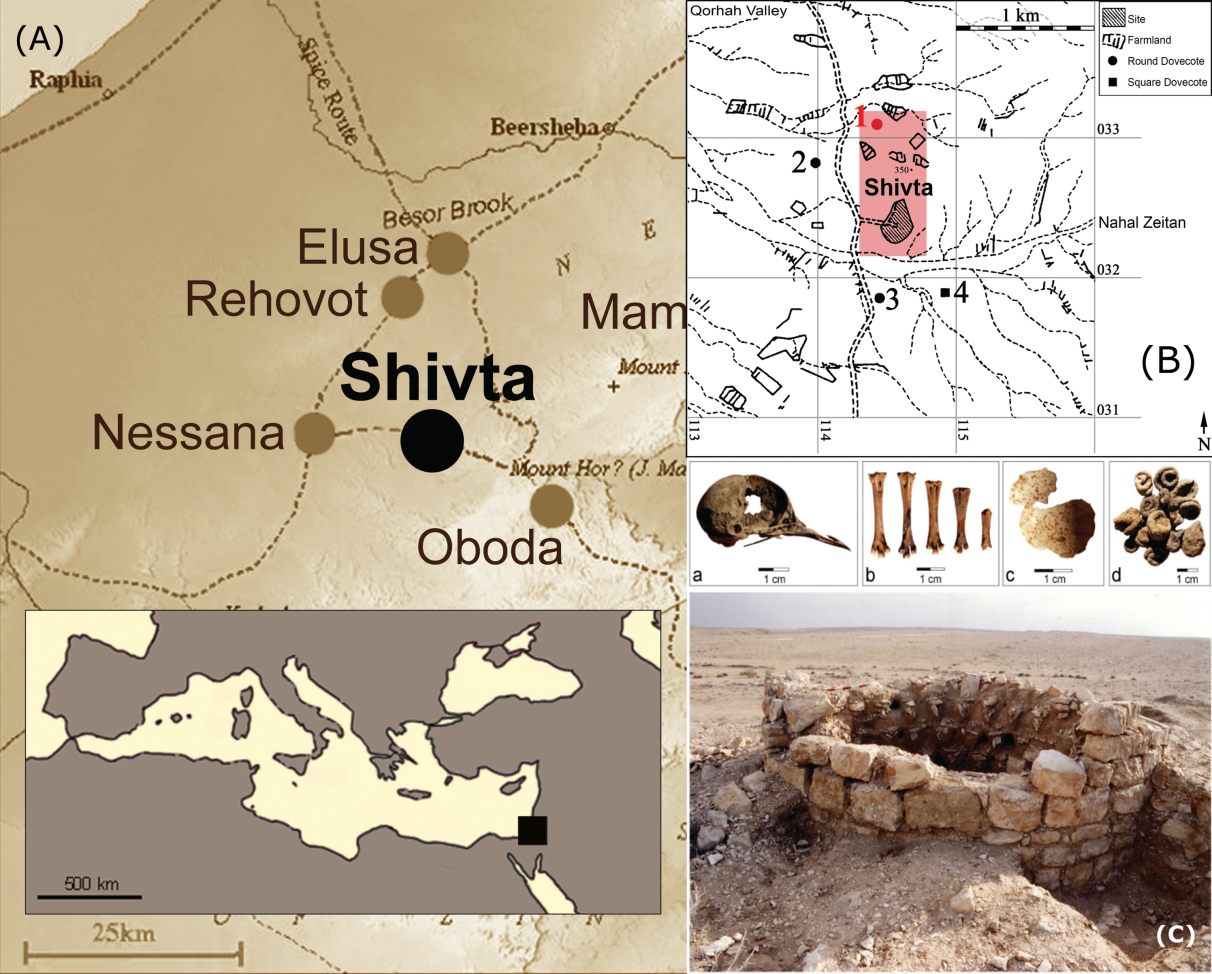
**Location map (A) for Shivta; aerial photograph and plan (B) of the site; pigeon tower during excavations (C); and a pigeon skull (C-a), bones (C-b), egg shell fragments (C-c) and droppings (C-d) from Shivta.** Photographs by Y. Tepper; maps drawn by Anat Regev-Gisis.

Against this background, we discuss a unique archaeological context dated to the 6^th^ century CE, in which catastrophic mortality in a pigeon tower allowed excellent preservation of numerous bone specimens from a single pigeon flock, including articulated individuals and a complete skull. The pigeon tower has been part of an intensively cultivated hinterland of the desert Byzantine settlement of Subeita (present day Shivta, western Negev, Israel; Fig 1)[7], which specialized in viticulture for production of the world-famous Gaza wine. It has been suggested [8] that the numerous pigeon towers dotting the settlement’s immediate vicinity supplied fertilizer for the region’s prohibitively poor soil, and have therefore served as a crucial component of Shivta’s agricultural system.

This study focuses on the Shivta archaeological pigeon flock and examines whether directed selection was taking place as the result of human breeding or, alternatively, whether stabilizing selection by the marginal environment was more important in determining its metric characteristics. In the first case, we would expect evidences for derived cranial and post-cranial morphology of the Shivta dovecote pigeons in relation to wild conspecifics (*Columba livia palaestinae*) [9]; and in the second case we would look for possible reduction of body mass [10], which may have been naturally selected for by the desert environment where carrying capacity is low and foraging ranges are correspondingly long. We expect the hypotheses on human as opposed to environmental selection to be mutually exclusive: human breeding for meat or sport, representing intensive management, would require isolation from external selective pressures leading to derived morphology; extensive *management*, in which humans afford only shelter and foraging opportunities, would result in exposure to environmental pressures such as climatic extremities and predation, selecting against excessive size or extraordinary proportions that are the aims of breeding. In other words, intensive management would result in directed selection away from the wild *Columba* morphotype while extensive management would result in stabilizing selection for the wild morphotype. Multivariate analysis of the archaeological remains in relation to recent reference specimens from different breeds and to archaeological pigeon specimens from the region should be able to distinguish directed (human induced) as opposed to stabilizing (environmentally induced) selection on pigeon morphology.

## Methods

Pigeon bones from Shivta were recovered using a high-resolution excavation protocol from the ruined pigeon tower dated radiometrically to the late sixth century CE [11]. The remains comprise of 143 identified bones accumulated as the result of a catastrophic mortality event. All the complete adult bones were measured using calipers to the nearest 0.1 mm, following von den Driesch [12] (SI 1).

Shivta pigeon morphology was studied in comparison to pigeons measured by the first author in the birds’ collection of the British Museum of Natural History in Tring, UK (S1 File; the data include measurements of the skeletons prepared by Darwin for his study on pigeon variations under domestication). The recent pigeons from Tring were divided into groups based on Darwin’s typology [13]:

- Pouters
- Runts, Barbs and Carriers
- Jacobines, Tumblers and Skimmers;
- Nuns, Laughters, Spots and other breeds that are morphologically like wild pigeons.

These were supplemented by three additional categories to accommodate specimen labels of Tring collection:

W : Pigeons labeled as “wild”;
F : Pigeons labeled as “feral”;
R : Racing pigeons.

Analysis of cranial size and shape relied on Discriminant Linear Analysis (LDA) of four measurements of recent pigeons (GL, SBO, CBL and GB), with the complete skull from Shivta coded as a “mystery specimen” [14]. The analysis focused on the skull since this is expected to show the highest variability between breeds [15]. In addition, Principal Component Analysis (PCA) was carried out to seek differences between post-cranial bones of pigeon groups based on 11 length measurements taken for each of 43 recent pigeon specimens selected for their relative completeness; single missing values were replaced by column averages. The Shivta pigeons’ measurements were incorporated into the analysis as mean values of all adult specimens. The first principal component (PC1), representing size, was regressed on wing length (sum of humerus, ulna and carpometacarpus measurements) and sternum length (LC) to examine allometry in these elements, which provide the lift and power stroke in flight.

**Fig 2:**
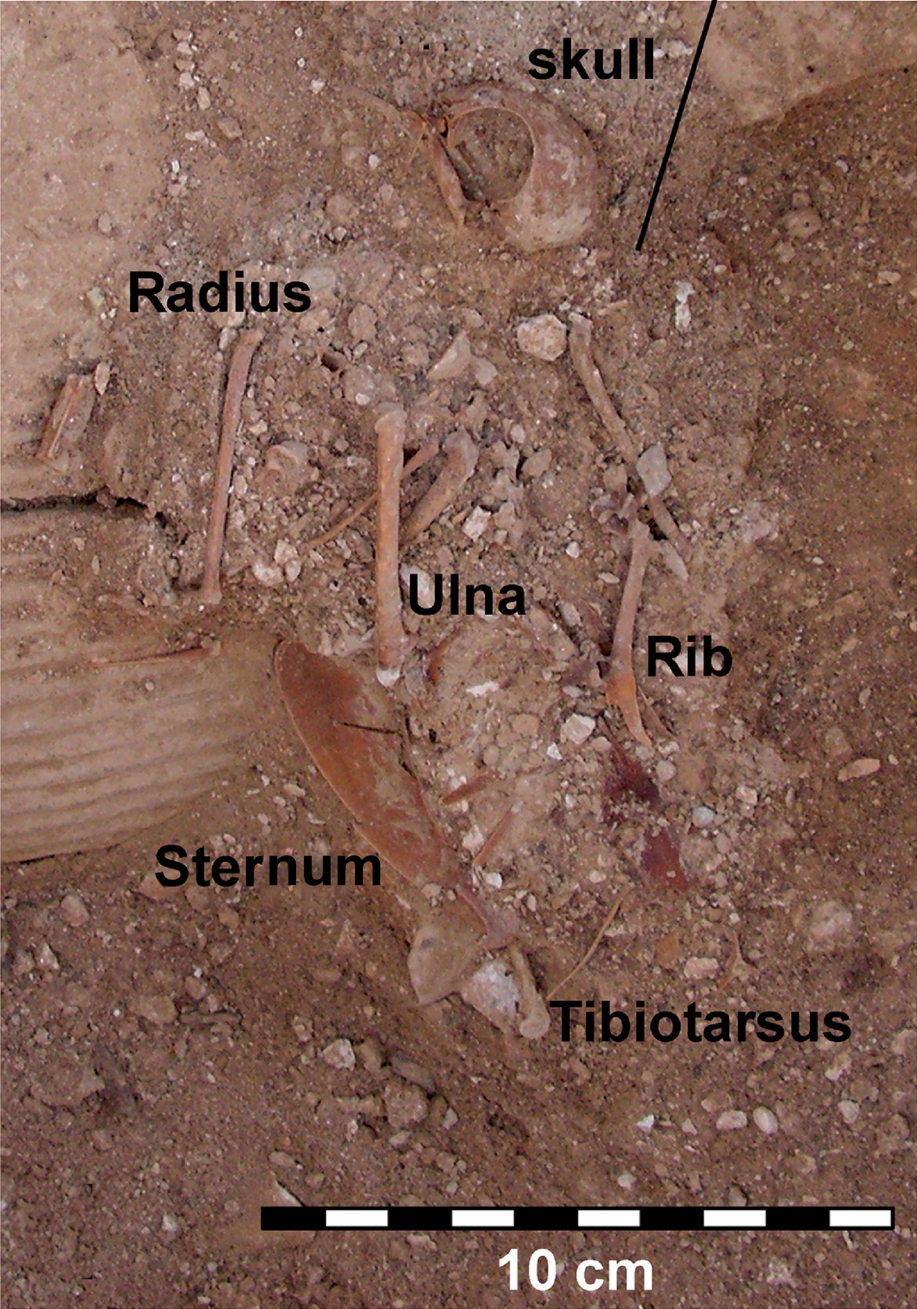
An articulated pigeon skeleton from Saadon. Photographed by Y Tepper.

Finally, the pigeons from Shivta were metrically compared to archaeological pigeons from other sites that yielded measurable remains: (1) Saadon, which is another Negev Byzantine assemblage, only more partial in terms of sample size and the measurements it provided (Fig 2)[16]; (2) Ramat Rahel, which represents a (radiocarbon dated) fourth century BC pigeon habitation in a cave near Jerusalem; (3) a single specimen from Qumran Cave 24 [17], representing a Neolithic (9^th^ millennium BC) pigeon from the region of Jericho in the Judean Desert, and (4) six pigeon measurements from European Upper Palaeolithic (ca. 40–20 kya) caves: Grotte du Lazaret [18] and La Grotte de la Hortus [19], both cited in [20]. The specimens were compared using log-size index [21] using the mean of the wild pigeon group as a benchmark. A Kruskal-Wallis Test for medians, a non-parametric alternative to ANOVA, was carried out to compare the log-size transformed data (not normally-distributed for the sample from Shivta; Shapiro-Wilk W = 0.95, P = 0.002). Pairwise comparisons employed non-parametric Mann-Whitney tests with bootstrap resampling (N = 9999 repeats) [22].

Statistical analyses were carried out in PAST 13.14 [23].

## Results

**Fig 3:**
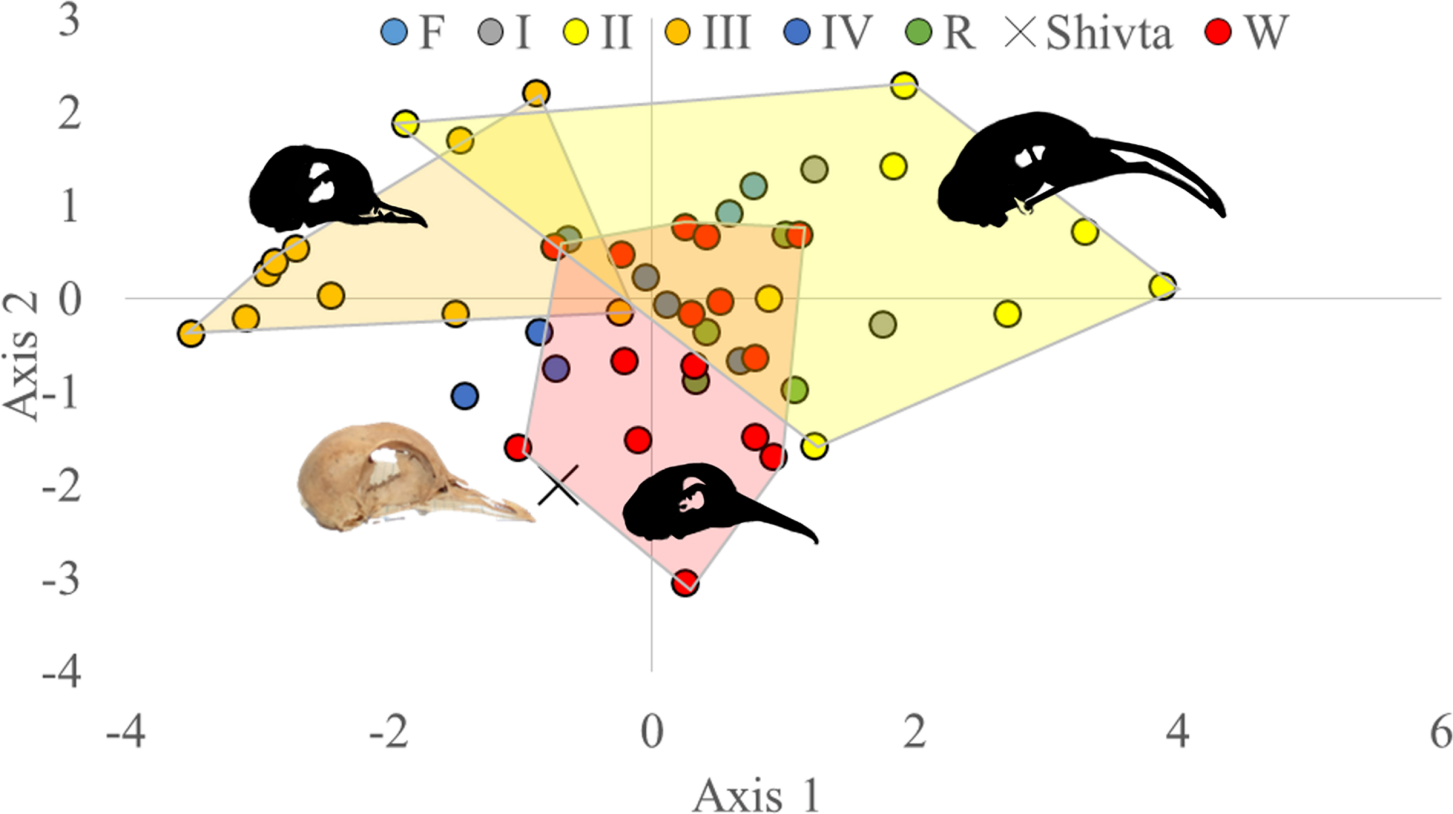
Linear discriminant analysis of four skull dimensions (see methods) and interpretive clustering illustrated by polygons. Skull illustrations redrawn by Anat Regev-Gisis from Darwin 1998; Photograph of the Shivta pigeon skull by N Marom (not to scale). Axis 1 explains 74% of the variability and Axis 2 13%.

Linear Discriminant Analysis of skull dimensions shows three relatively distinct clusters (Fig 3): one centered in the upper left quadrant of the chart, comprising of the short-faced breeds of Group III; one in the center of the chart, comprising of wild (Group W) and toy (Group IV) specimens; and other groups comprising of larger pouters (Group I), barbs, runts, carriers (Group II) and racing pigeons (Group R) with positive first and second axis values. The Shivta skull is classified by the analysis as belonging in Group IV (toy breeds), or is, in other words, similar to a wild pigeon.

**Fig 4:**
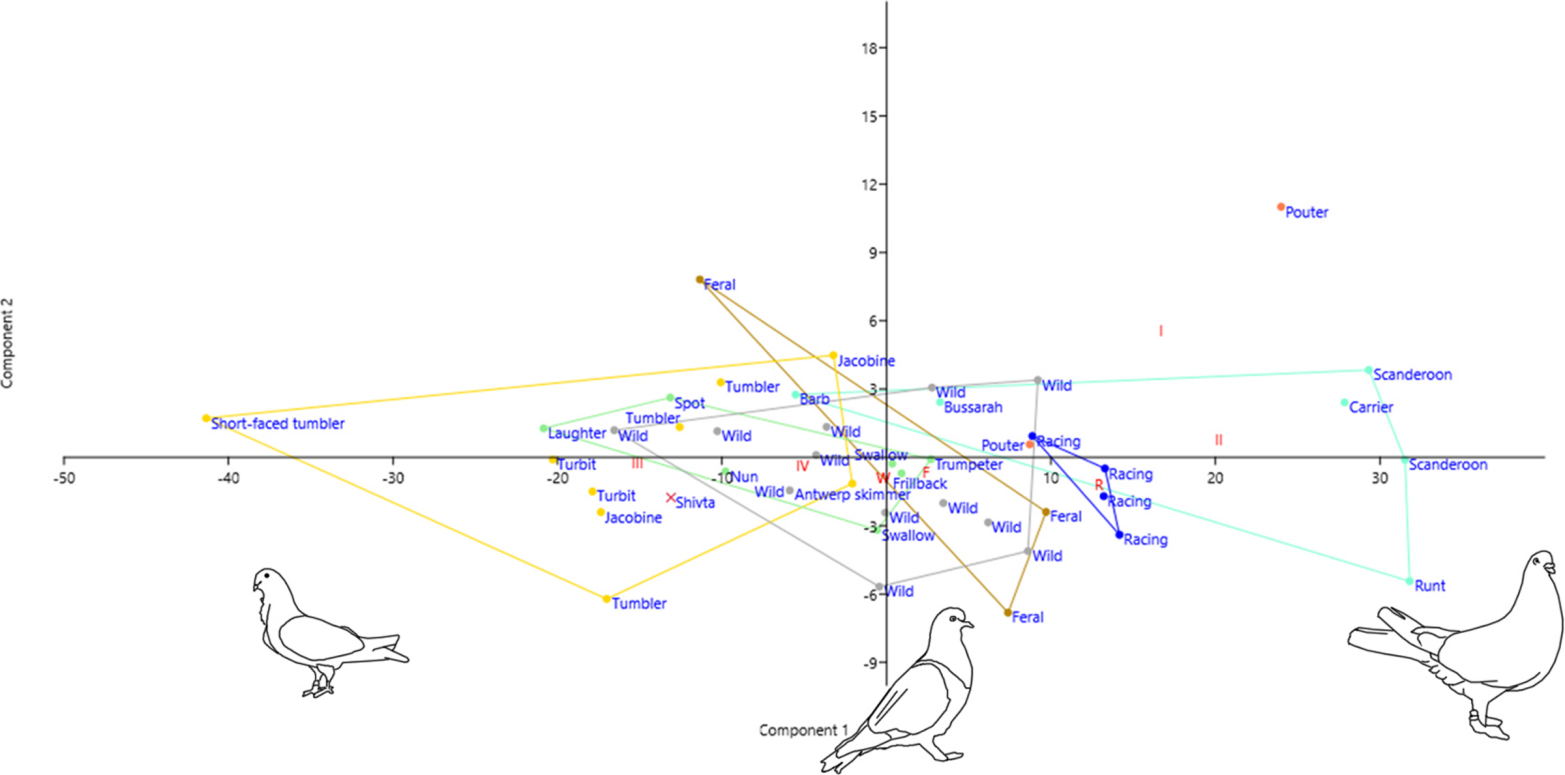
PCA of 14 measurements on 46 recent pigeon specimens and an “average” Shivta pigeon. Axis 1, explaining 88% of the variability, is interpreted as size; Axis 2 accounts for 4% of the variability. Pigeon illustrations by Anat Regev-Gisis.

Principle Component Analysis places the Shivta pigeons in the overlap between wild pigeons, Group III, and Group IV along the first principal component. The first principal component is very dominant, explaining 88% of the variability in the data. In its positive extreme are Carriers and Runts, and in its negative extreme Tumblers, suggesting that the variability it describes is in body-size. Since the second principal component explains only 4% of the variability observed in the data, it is insignificant and consequent decompositions of the covariance matrix can be ignored. The regression of body-size (PC1) on wing and sternum lengths (Fig 4) yields no indications for allometric growth in the Shivta pigeons, which are close to the lines of best fit.

**Fig 5:**
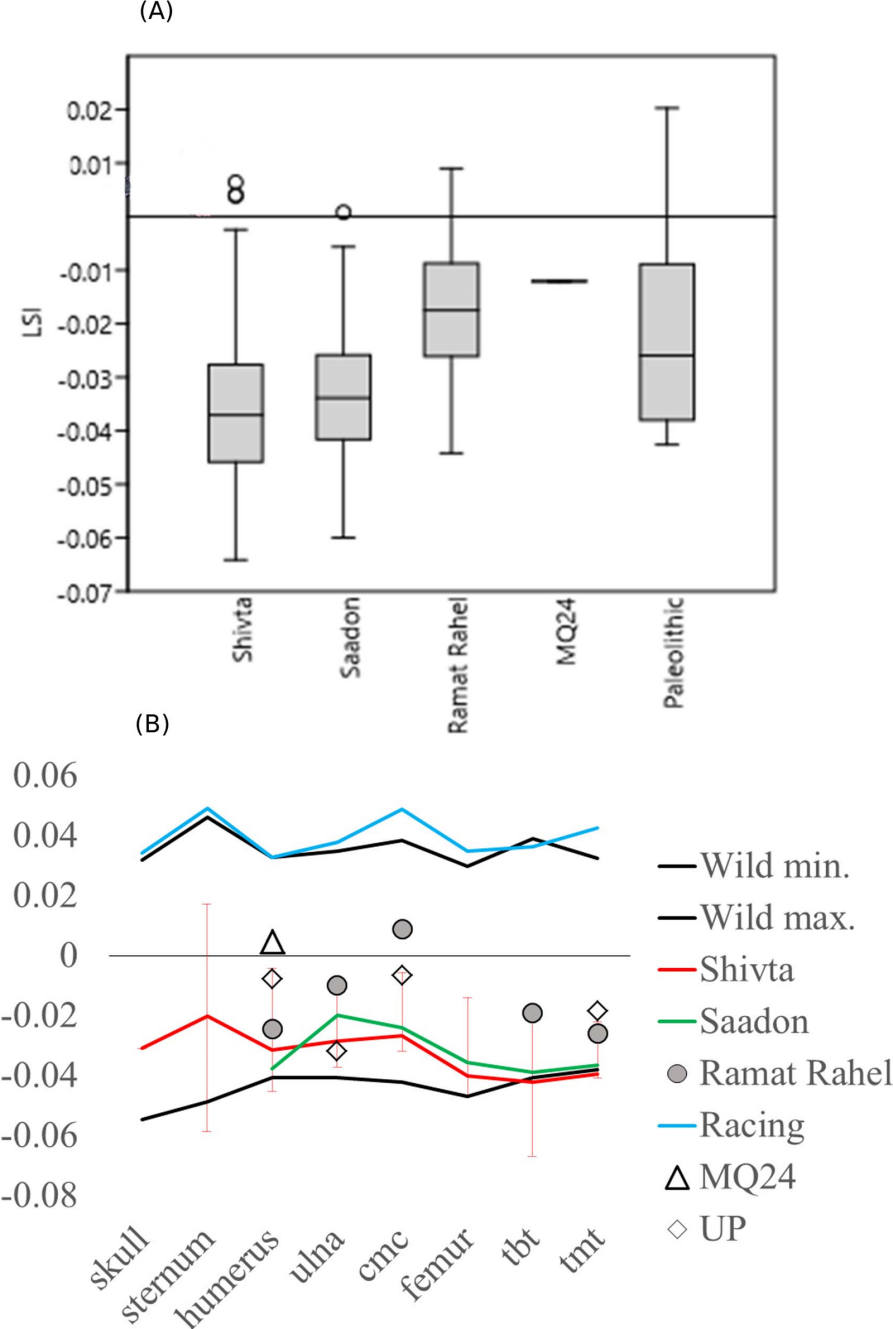
Log size index analyses. The abscissa represents the mean from each element of the wild pigeons in the BMNH collections. (a) averages and ranges of the LSI for the Shivta pigeons and reference groups; (b) lsi for all elements pooled for archaeological pigeons.

Log-size ratio analysis of the Shivta measurements (N = 108) shows them to be smaller than the mean size of recent wild pigeons in the Tring collection (Fig 5). Comparison with other sites shows very close similarity in body-size to the contemporary pigeons from Saadon; but both Byzantine pigeon groups are smaller than the wild archaeological population represented in Ramat Rahel (Kruskal-Wallis H_C_ = 13.51, P = 0.001). Although the finds from Neolithic and Paleolithic contexts are few, it appears from the chart that Ramat Rahel, Qumran Cave and the Upper Paleolithic European contexts represent similar populations with respect to body-size.

In summary, body-size appears to be the major component of variability between pigeon groups. LDA analysis of skull dimensions places the Shivta pigeons in Darwin’s Group IV, which comprises of birds that are not morphologically different from wild pigeons; multivariate analysis including post-cranial measurements places the Shivta pigeons in the lowest size range of wild pigeons and within the size range of small domestic breeds. Comparison with available archaeological biometric data shows that the Shivta pigeons are as large as those of the contemporary and nearby site of Saadon, but significantly smaller than archaeological wild pigeons from Ramat Rahel, near Jerusalem (Mann-Whintney U = 271.5; Permutation P = 0.0004). These later pigeons are of a size similar to Neolithic and Paleolithic wild pigeons from Israel and Europe.

## Discussion

Pigeon breeding can be expected to manifest in the archaeological finds through body-size and skull shape diversification from a wild phenotype. The larger breeds like carrier, racing, and meat pigeons were not kept in the Byzantine Negev, as body-size data clearly demonstrate. The rare skull from Shivta does not cluster with the short-faced breeds, but rather with a general wild phenotype, precluding the keeping of such fancy pigeons in the Negev pigeon towers. The metric evidences are therefore consistent with the presence in the Shivta tower of a population of small rock-pigeons.

The small body-size in relation to wild archaeological pigeons and to recent wild pigeons in the Tring collection is notable, and can perhaps be interpreted using Bergmann’s rule, which applies to *C*. *livia* [10]. Bergmann’s rule appears to reflect changes in body-size related to carrying capacity, which would explain the difference between Ramat Rahel (in the Mediterranean zone) and the desert sites. If this interpretation is correct, we can infer exposure of the Shivta pigeon population to environmental stress: they were not fed in an orderly way by humans typical for the keeping of dove cote pigeons, and quickly reverted to a feral/wild morphotype [24], typical of the plasticity in body-size observed in pigeons [25].

The pigeons kept in Shivta were therefore not kept for their meat or for sportive breeding, buffered from the environment by an intensive management regime. On the contrary, extensive management of wild rock pigeon populations that were afforded shelter and foraging opportunities by humans is suggested to have been practiced. Since breeding for meat or fancy were not practiced, a “secondary product”, fertilizer, may well have been the incentive for pigeon keeping, as was previously suggested based on agronomic considerations and historical and archaeological data [8]. Pigeon-raising in towers is still deeply rooted in the traditional subsistence agriculture in the rural villages of the Middle East. Pigeon towers are usually built in proximity to cultivated areas and the importance of pigeon manure cannot be underestimated. In many cases in the ancient Middle East it was a major source organic fertilizer available, primarily for annual crop farming, particularly irrigated crops and orchards [26]. Given its high nitrogen content, pigeon manure is especially effective in soils poor in minerals and organic matter, such as chalky and loess soils that cannot support intensive agriculture without proper fertilization.

Lack of environmental buffering by humans under the suggested extensive regime would probably leave other traces in the bioarchaeological record. For example, uniform plumage coloration is to be expected, since conspicuously colored or patterned birds would be easier targets for predation in a flock [27]. Genetic studies of the remains would therefore be indispensable in providing additional support to the extensive management hypothesis, over and beyond recent genetic work targeting pigeon breeding, feralization, and recent phylogeny [28, 29, 30, 31].

**Fig 6:**
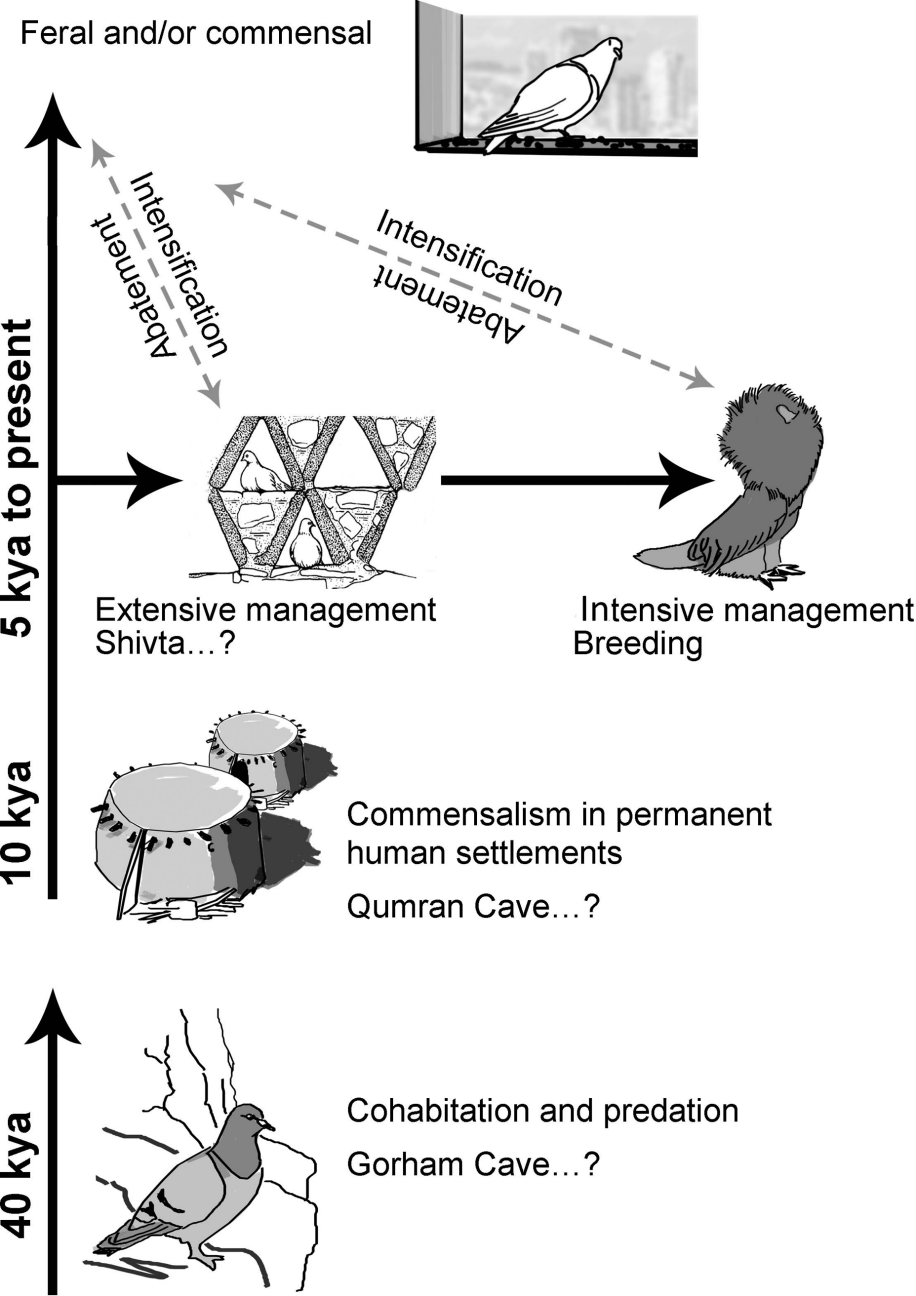
Schematic illustration of human-pigeon interaction through time. Drawings by Anat Regev-Gisis.

The process of pigeon domestication is poorly understood due to a nearly complete absence of bioarchaeological datasets. Conjecturally, the process could have begun as commensal relations first established in Pleistocene cave habitations shared by both taxa [32]; however, it is very difficult to tease apart predation on pigeons inhabiting caves at that period from sustainable co-habitation given the rarity of the finds and their susceptibility to diagenetic destruction. An incentive for true commensalism would have begun with agriculture, as human settlements provided a dense and constantly replenishing foraging patch for pigeons. A nice frame that support such a scenario comes from the Neolithic site of Qumran who is located near Jericho. From that time, when constant motivation to live and nest by humans was created, a Rubicon was crossed; pigeons were bound to human environments, drawn to it by foraging opportunities, and could be exploited with varying degrees of intensity [33] (Fig 6).

While in Rome large pigeons were kept for their meat in great numbers, in the desert frontier of later Antiquity we present evidences for extensive husbanding of pigeons, supporting an earlier suggestion for the use of pigeon secondary product: fertilizer [34, 35, 36]. Pigeons at this margin of the empire served as an essential component in a complex agricultural production system, and their extensive management system is certainly one of the clearer illustrations for sustainable technological ingenuity by which marginal environments can be utilized as lucrative “micro-ecologies” within an interconnected Mediterranean world. If fertilizing local soils by pigeon manure was essential for viticulture, viticulture provided the storable and redistributable commodity that could be traded for staples during bad years [37]; in a marginal zone, this risk-abating product meant long-term settlement viability. By understanding how the Shivta pigeons were managed we have advanced another step towards understanding human productivity and resilience strategy at the margin of the empire.

## Supporting information

S1 File Archaeological and recent pigeon measurements from the British Museum Tring collection (first tab) and by skeletal element, including archaeological specimens (following tabs). Measurements were taken by NM using analogic calipers to 0.1 mm, except when otherwise stated.
(XLSX)
